# Authenticity Identification and Quantitative Analysis of *Dendrobium officinale* Based on Near-Infrared Spectroscopy Combined with Chemometrics

**DOI:** 10.3390/foods15010121

**Published:** 2026-01-01

**Authors:** Zhi-Liang Fan, Qian Li, Zhi-Tong Zhang, Lei Bai, Xiang Pu, Ting-Wei Shi, Yi-Hui Chai

**Affiliations:** 1 School of Basic Chinese Medicine, Guizhou University of Traditional Chinese Medicine, Guizhou 550000, China; 2 Jiangsu Province Engineering Research Center of Classical Prescription, School of Pharmacy, Nanjing University of Chinese Medicine, Nanjing 210023, China; 3 School of Pharmacy, Guizhou University of Traditional Chinese Medicine, Guizhou 550000, China

**Keywords:** *Dendrobium officinale*, near-infrared spectroscopy, DD-SIMCA, PLSR

## Abstract

*Dendrobium officinale* is a valuable medicinal and edible homologous health food. It has immunomodulatory, antioxidant, and metabolism-regulating properties. However, its adulteration is widespread, seriously compromising product quality and safety. Traditional adulteration detection methods are complex, costly, and time-consuming, making it urgent to establish a rapid and non-destructive detection approach. This study developed a rapid identification and quantification method for adulterated D. officinale. The method combined near-infrared (NIR) spectroscopy with data-driven soft independent modeling of class analogy (DD-SIMCA) and partial least squares regression (PLSR) models. PCA, PLS-DA, and OPLS-DA were first used to visualize sample clustering and group differences. DT, SVM, ANN, and NB were used for classification. DD-SIMCA and PLSR were used for one-class modeling and quantitative analysis. Raw spectral data were preprocessed using multiplicative scatter correction (MSC), the standard normal variate (SNV), the first derivative, and Savitzky–Golay smoothing. In the identification analysis, the DD-SIMCA model achieved 100% sensitivity and 100% specificity in the validation set. Its overall accuracy in the independent test set was 99.2%, demonstrating excellent discrimination performance. In addition, SVM combined with NIR also achieved good accuracy. In the quantitative analysis of adulteration, the PLSR model predicted different adulteration levels. Most calibration and validation sets showed R^2^ values above 0.99 and RMSE values below 0.05, indicating excellent predictive performance. The results indicate that NIR combined with DD-SIMCA and PLSR can achieve rapid identification and accurate quantification of adulterated *D. officinale* samples. This approach provides strong support for quality control and regulatory supervision of high-value health foods.

## 1. Introduction

In China and other Southeast Asian countries, plants of the genus *Dendrobium* hold dual value as both food ingredients and traditional herbal medicines. Among them, *Dendrobium officinale* Kimura et Migo (DKM) is recognized as one of the most precious Chinese medicinal materials, and it is widely distributed across southern China and Southeast Asia [1,2]. According to the *Pharmacopoeia of the People’s Republic of China*, there are two independent entries for *Dendrobium*—“Shi Hu” and “Tie Pi Shi Hu”. The “Tie Pi Shi Hu” entry exclusively lists DKM as its source plant, whereas “Shi Hu” encompasses *Dendrobium nobile* Lindl. (DL), *Dendrobium fimbriatum* Hook. (DH), and several other congeneric species. Modern pharmacological studies have demonstrated that DKM possesses several biological activities, including immunomodulation, antioxidant effects, and mucosal protection [3,4]. These properties support its wide application in anti-aging, adjuvant diabetes therapy, and gastrointestinal regulation.

In recent years, DKM has been increasingly developed into various products, including health foods and skincare formulations, across Asia, reflecting its substantial economic value. However, the widespread adulteration of DKM—primarily involving the mixing of other *Dendrobium* species—has emerged as a significant challenge. Currently, there is no conclusive evidence confirming whether these adulterants provide the same therapeutic efficacy as DKM. Consequently, such adulteration not only disrupts market order but also undermines the rights of consumers and patients. Therefore, the development of a rapid and accurate authentication strategy for DKM is urgently needed. Nevertheless, because these species are closely related to DKM, their morphological characteristics are highly similar, making visual discrimination extremely difficult. Furthermore, DKM used in health products is commonly processed into powder, which further complicates authenticity identification.

In current research on the authenticity and adulteration identification of DKM, multiple-source chromatographic platforms such as Near-Infrared (NIR) Spectroscopy, Laser-Induced Breakdown Spectroscopy (LIBS), and UHPLC-MS/MS are widely applied. These methods, combined with chemometrics and machine learning approaches like PLS-DA and SVM, have significantly improved the accuracy of classification and identification. Relevant studies show that the prediction accuracy for *Dendrobium* species discrimination is close to 90% when combining NIR-Hyperspectral Imaging, feature selection, and SVM [5]. Furthermore, LIBS can achieve rapid differentiation of *Dendrobium* and its closely related species by analyzing differences in elemental composition or characteristic peaks [6]. Meanwhile, mass spectrometry-based and deep learning models for origin and quality traceability have further demonstrated their strong applicability for complex pattern recognition tasks, providing more reliable technical support for DKM adulteration monitoring [7]. In addition, NMR metabolomics and qNMR fingerprinting have been widely applied in Dendrobium species differentiation and metabolite profiling due to their clear structural resolution and high reproducibility [8,9,10]. However, their discriminative ability becomes limited when dealing with complex matrices, low-abundance constituents, or multi-source commercial materials. Thus, despite the advantages of multi-spectral platforms, current studies still lack targeted investigation specifically focusing on DKM itself [11,12].

NIR is an analytical technique that involves the interaction between light in the wavenumber range of approximately 13,000–4000 cm^−1^ (corresponding to wavelengths of 780–2500 nm) and matter. It primarily reflects the overtone and combination vibration absorptions of hydrogen-containing functional groups, such as C–H, O–H, and N–H [13]. Compared with conventional chromatographic or mass spectrometric techniques, NIR analysis offers advantages such as rapid detection, non-destructive testing, simple operation, and environmental friendliness. Consequently, NIR has been widely applied in compositional analysis and quality control of foods, Chinese herbal medicines, and agricultural products. For authenticity identification, NIR combined with multivariate analysis and machine learning algorithms can effectively distinguish adulterated samples from genuine ones, making it particularly suitable for non-destructive detection of powdered substances. Representative applications include identifying adulteration in *Arnebia* Radix, detecting non-target crop powders in Tartary buckwheat flour, and recognizing starchy adulterants in *Panax notoginseng* powder [14,15,16].

However, these conventional adulteration detection methods still present clear limitations. They depend heavily on prior knowledge of adulterants and often exhibit low sensitivity to minor adulteration. In contrast, data-driven soft independent modeling of class analogy (DD-SIMCA) offers distinct advantages for such tasks. This class modeling approach explores the intrinsic structure of the data using principal component analysis. It constructs a feature space based solely on authentic samples, without requiring prior knowledge of adulterants. Consequently, it can effectively identify unknown adulteration. By focusing on intrinsic intra-class features, DD-SIMCA minimizes model fluctuations caused by variations in adulteration type or proportion. This method thus provides a novel and efficient technical pathway for authenticity assessment of *D. officinale*.

## 2. Materials and Methods

### 2.1. Sample Collection and Preparation

Ten batches of *Dendrobium officinale* Kimura et Migo (DKM) were collected, along with one batch each of *D. loddigesii* (DL), *D. hancockii* (DH), DKM leaves (DKMLs), bamboo, and corn. Bamboo powder and corn powder were purchased from local markets (Guiyang, Guizhou Province, China), while the remaining samples were sourced from the Bozhou market in Anhui Province, China. All plant materials were authenticated as genuine by Professor Qingwen Sun from Guizhou University of Traditional Chinese Medicine.

Each sample was finely ground and sieved through a No. 5 sieve (80 mesh) to ensure a consistent particle size. Representative adulterants are shown in Supplementary Figure S1. Based on the experimental design, the materials were divided into three categories: pure DKM samples, adulterated DKM samples (with 20%, 40%, 60%, and 80% adulteration levels), and pure adulterant samples (bamboo, corn, DL, DH, and DKML). A total of 275 samples were prepared, and detailed sample information is provided in Supplementary Table S1.

### 2.2. NIR Spectral Acquisition

Diffuse reflectance near-infrared spectra were acquired using the Fourier-transform NIR spectrometer (Thermo Fisher Scientific, Waltham, MA, USA) equipped with an integrating sphere. Spectra were collected over the range of 10,000–4000 cm^−1^ with a resolution of 8 cm^−1^, resulting in 1557 spectral data points per sample. The data were expressed as log(1/R), where R is the relative reflectance.

For each measurement, approximately 3 g of powder was placed in a sample cup, gently shaken, and leveled to ensure uniform packing. Sixty-four scans were averaged to enhance the signal-to-noise ratio and minimize random error. All spectral measurements were conducted under consistent laboratory conditions to reduce environmental variability.

### 2.3. Exploratory Data Analysis

To explore sample distribution patterns and potential clustering, multivariate visualization techniques were applied. These included Principal Component Analysis (PCA-X), Partial Least Squares Discriminant Analysis (PLS-DA), and Orthogonal Partial Least Squares Discriminant Analysis (OPLS-DA) [17]. PCA-X, an unsupervised method, was used to examine the overall variance structure of the spectral data. In contrast, PLS-DA and OPLS-DA, both supervised methods, incorporated group information to enhance class discrimination.

PLS-DA was used to construct an optimal regression model between spectral variables and categorical sample groups. OPLS-DA further removed orthogonal components unrelated to class differences, thereby improving interpretability and predictive performance [18]. Model quality was assessed using R^2^ (explained variance) and Q^2^ (predictive ability) obtained through cross-validation.

### 2.4. Chemometric Classification Modeling

Supervised machine learning methods were used to construct classification models capable of distinguishing genuine DKM from adulterated samples. Four commonly used algorithms—Decision Tree (DT) [19], Support Vector Machine (SVM) [20], Artificial Neural Network (ANN) [21], and Naive Bayes (NB) [22]—were implemented in MATLAB R2022b (MathWorks, Natick, MA, USA) using the Classification Learner Toolbox. The NIR spectral data served as input variables, while class labels (genuine/adulterated) were used as outputs.

A 10-fold cross-validation strategy was adopted to enhance model robustness and reduce overfitting. Classification accuracy and confusion matrices were used as evaluation metrics to compare the discriminative performance of different algorithms.

### 2.5. DD-SIMCA Single-Class Modeling

The DD-SIMCA is a single-class classification algorithm that focuses on modeling the intrinsic structure of genuine samples to enable sensitive detection of deviations, such as adulteration. In this study, DD-SIMCA was implemented following a standard three-step procedure [23]. First, PCA was performed on the calibration set to decompose the spectra and extract principal components representing the major variance structure. Next, score distance and orthogonal distance for each calibration sample were calculated based on the PCA scores. These distances were combined with data-driven estimates of scaling factors and degrees of freedom to obtain the total distance. Finally, the acceptance region and classification thresholds for the positive class were established, such that a new sample falling within this region would be identified as genuine.

To ensure robust evaluation and model generalization, the Kennard-Stone algorithm was applied to partition the dataset into three subsets—the training, validation, and independent external test sets—using a 6:2:2 ratio [24]. The Validation Set (20%) was strictly dedicated to hyperparameter optimization, thus preventing data leakage. Crucially, the Independent External Test Set (20%) was reserved and completely untouched throughout the entire modeling and parameter tuning process. This set was used only once for the final, unbiased evaluation of the model’s predictive performance, thereby ensuring the reliability and generalizability of the reported results [25]. To strictly control the misclassification risk of genuine samples as adulterated, the Type I error rate and outlier significance level were both set to 0.01. The optimal number of principal components (PCs) was determined by adjusting hyperparameters on the validation set to achieve the best balance between sensitivity and specificity.

The performance of the DD-SIMCA model was evaluated using accuracy, specificity, and sensitivity. Accuracy refers to the proportion of correctly classified samples among all samples; specificity indicates the proportion of correctly identified negative-class samples (adulterated) among actual negative-class samples; and sensitivity represents the proportion of correctly classified positive-class samples (genuine) among actual positive-class samples. These metrics were calculated based on the counts of correctly and incorrectly classified genuine and adulterated samples, providing a comprehensive assessment of the model’s discriminative ability.

### 2.6. PLSR Quantitative Modeling

Partial Least Squares Regression (PLSR) is a multivariate regression method that reduces the dimensionality of spectral and dependent variables while maximizing their covariance, suitable for high-dimensional and collinear data [26]. In this study, PLSR was applied to quantitatively predict adulteration ratios in DKM samples based on NIR spectra.

First, raw spectral data were preprocessed to reduce noise, correct baseline drift, and minimize scattering effects. Specifically, fourteen preprocessing combinations were tested, including 1st and 2nd derivatives (1st Der & 2nd Der), Savitzky–Golay (SG) smoothing, multiplicative scatter correction (MSC), standard normal variate (SNV), and their combinations, in order to enhance spectral features and improve model performance [27].

Then, the data were divided into calibration and prediction sets using the Kennard–Stone algorithm at a 7:3 ratio. Subsequently, the number of latent variables (LVs) was determined via 10-fold cross-validation, by selecting those corresponding to the minimum root mean square error of cross-validation (RMSECV).

To comprehensively evaluate the model’s performance, several metrics were calculated, including the coefficient of determination of the calibration set (R^2^c), coefficient of determination of the prediction set (R^2^p), root mean square error of calibration (RMSEC), root mean square error of prediction (RMSEP), mean absolute error (MAE), and relative percent deviation (RPD), which collectively reflect fitting accuracy, predictive ability, and model generalization.

Finally, for each DKM–adulterant pair, the PLSR model with the optimal preprocessing strategy was selected to quantify adulteration content, ensuring reliable prediction and supporting authenticity assessment and quality control.

### 2.7. Data Analysis

DD-SIMCA and PLSR modeling procedures were performed in MATLAB R2022b. Spectral preprocessing, PCA, PLS-DA, and OPLS-DA analyses were conducted using SIMCA 14.1 (Umetrics, Umeå, Sweden).

## 3. Results

### 3.1. Near-Infrared Spectral Analysis

NIR spectroscopy provides characteristic absorption bands that arise from the first overtones and combination vibrations of molecular functional groups. In this study, the representative absorption peaks were mainly located in the ranges of 7075–6600 cm^−1^, 5200–5100 cm^−1^, and 4800–4600 cm^−1^. Specifically, the band at 7075–6600 cm^−1^ is generally attributed to the first overtone of O–H stretching vibrations. Its pronounced intensity suggests a high abundance of hydroxyl-containing compounds, such as polyphenols and alcohols [28]. The absorption band observed at 5200–5100 cm^−1^ may originate from the combination of O–H stretching and C–H bending vibrations, reflecting the synergistic vibrational features of hydroxyl and alkyl groups in sugars or polysaccharides [29]. Meanwhile, the band at 4800–4600 cm^−1^ is commonly associated with the first overtone of C–H stretching vibrations and O–H combination frequencies in water, and it is often used to characterize trace moisture or bound water in plant samples [30].

A comparison of the average spectra between DKM and adulterated samples revealed subtle but discernible differences in absorption intensity, peak shape, and fine spectral structure at specific wavenumbers, particularly near 5600 and 5100 cm^−1^. These variations likely reflect compositional differences, including the relative contents of polysaccharides, alcohols, and proteins, as well as structural differences such as hydroxyl positioning, chain length, or molecular conformation. However, the complexity of the spectral signals makes it difficult to achieve accurate classification based solely on direct spectral inspection. Therefore, multivariate statistical analysis and machine learning methods were applied to further explore and extract latent discriminative information from the high-dimensional data.

It should be noted that raw NIR spectra of powdered plant materials are often affected by baseline drift, scattering effects, and random noise. These interferences can obscure subtle chemical information and reduce model robustness [31]. To address these issues, spectral preprocessing techniques were applied, including 1st Der, MSC, and SG filtering. These methods enhance spectral resolution, reduce scattering, and correct baseline fluctuations, respectively [32,33].

**Figure 1:**
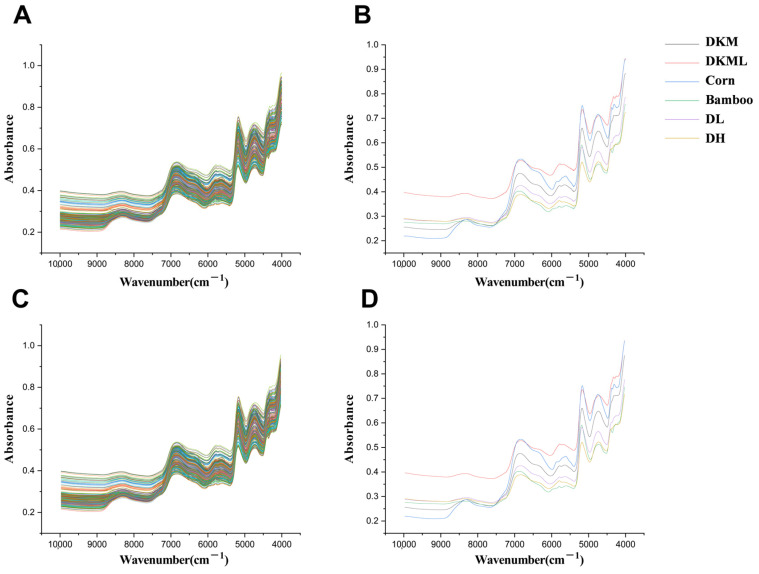
(**A**) Average raw NIR spectra of all powder samples. (**B**) Average raw NIR spectra of individual pure powders. (**C**) Optimized NIR spectra of all powder samples after SG pretreatment. (**D**) Optimized NIR spectra of individual pure powders after SG pretreatment.

After evaluating model performance under different preprocessing strategies, only minor differences were observed in R^2^ and Q^2^ values across methods. This indicates comparable feature representation and robust support for subsequent class discrimination. Nevertheless, SG filtering yielded the best overall performance, indicating its superior ability to preserve informative spectral details while reducing noise (Figure 1). This result may be attributed to SG filtering’s ability to effectively smooth random noise while preserving key spectral features, such as peak shapes and local variations. Such properties promote more reliable feature extraction and ultimately improve both classification and quantitative modeling accuracy [34].

### 3.2. Discriminant Analyses Using PCA, PLS-DA, and OPLS-DA

To further investigate the spectral differences between genuine DKM and adulterated samples, PCA, PLS-DA, and OPLS-DA models were constructed, and their discriminative performances were systematically evaluated.

In the unsupervised analysis, PCA was employed to reveal the overall distribution structure of the samples. Only four PCs were extracted, explaining 99.9% of the total variance, indicating that the dataset was highly compact and the selected PCs sufficiently captured its structure. To assess intra-class consistency, separate PCA models were constructed for six categorical subsets. Each subset exhibited high goodness of fit and predictive ability (R^2^X > 0.95 and Q^2^ > 0.994), reflecting the strong spectral homogeneity within each group rather than indicating superior model robustness. However, as an unsupervised method, PCA mainly reveals overall trends and clustering patterns. Its capacity to achieve complete separation among categories is limited, which makes precise classification impossible.

For supervised modeling, PLS-DA was first applied. The model achieved excellent goodness of fit (R^2^X = 1.000) when eight PCs (A = 8) were extracted, yet its predictive ability remained moderate (Q^2^ = 0.793).This result indicates that PLS-DA alone cannot achieve complete discrimination between adulterated and genuine samples. By further incorporating orthogonal signal correction, the OPLS-DA model was constructed, which substantially improved predictive performance. With eleven PCs (A = 11), OPLS-DA achieved the same R^2^X value (1.000) while increasing Q^2^ to 0.882, reflecting enhanced generalization ability and model stability.

**Figure 2:**
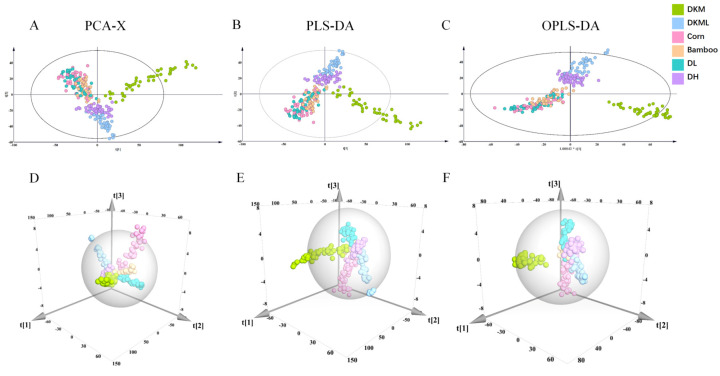
NIR multivariate analysis results: (**A**,**D**) PCA-X; (**B**,**E**) PLS-DA; (**C**,**F**) OPLS-DA.

Although OPLS-DA more effectively removes variations irrelevant to class discrimination compared with PLS-DA, traditional spectral analysis and multivariate modeling methods still struggle to achieve full separation between adulterated and genuine samples. These results underscore the necessity of integrating advanced optimization algorithms and efficient machine learning approaches to improve classification accuracy (Supplementary Table S2, Figure 2).

### 3.3. Discriminant Analysis Using Machine Learning Algorithms

Given that traditional chemometric approaches (PCA, PLS-DA, and OPLS-DA) were insufficient to achieve complete separation between genuine and adulterated DKM samples, we further constructed four supervised machine learning models—DT, SVM, ANN, and NB. These algorithms offer distinct advantages for handling complex, high-dimensional datasets: SVM maximizes class separation by constructing an optimal hyperplane; ANN mimics neuronal information processing, effectively capturing nonlinear feature patterns; DT offers an interpretable, hierarchical decision structure; and NB applies probabilistic reasoning under the assumption of conditional feature independence, enabling fast and computationally efficient classification [35].

**Figure 3:**
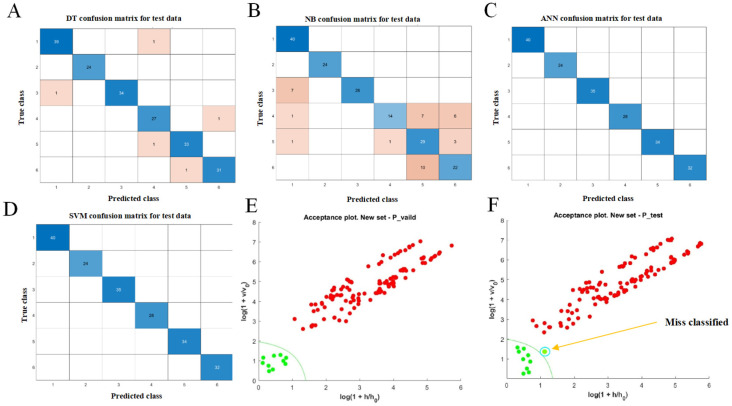
Classification analysis based on NIR spectra: (**A**) DT test confusion matrix; (**B**) NB test confusion matrix; (**C**) ANN test confusion matrix; (**D**) SVM test confusion matrix; (**E**,F) DD-SIMCA acceptance plots. In (**A**–**D**), blue cells represent correctly classified samples, while pink cells indicate misclassified samples. In (**E**,**F**), red markers represent samples identified as adulterated, and green markers represent samples identified as genuine by the model.

The classification results on the test set demonstrated that SVM achieved 100% accuracy, followed by ANN at 100%, DT at 97.4%, and NB at 81.3% (Supplementary_Table S3, Figure 3A–D). SVM correctly classified all samples, indicating excellent generalization ability and strong adaptability to spectral discrimination tasks. ANN also performed robustly, effectively identifying subtle differences between genuine and adulterated samples. In contrast, DT and NB exhibited lower accuracy, likely due to their sensitivity to noise and limited capacity to model complex relationships within high-dimensional spectral features.

These findings highlight that machine learning-based approaches, particularly SVM and ANN, outperform traditional multivariate statistical methods in authenticity identification of DKM. Nevertheless, potential limitations remain: misclassification or overfitting may arise when models encounter previously unseen adulterant types, highly imbalanced datasets, or extreme adulteration ratios. Consequently, integrating single-class or one-class modeling frameworks may further strengthen model robustness and enhance reliability in practical authentication scenarios.

### 3.4. Classification Performance of the DD-SIMCA Model

Although multi-class models such as SVM show high classification accuracy, they depend on comprehensive training data covering all classes. In practical scenarios—where genuine samples far outnumber known adulterants or adulterant types are unknown or variable—such models may show reduced robustness [36]. To address this limitation, a single-class discriminant approach, DD-SIMCA, was employed to construct a robust model based exclusively on genuine samples. Traditional multi-class classification methods have limited adaptability under such conditions, particularly when class imbalance or unknown classes arise [37].

The DD-SIMCA model establishes an exclusive spectral feature space for the target class, enabling sensitive and efficient detection of samples that deviate from the genuine class without requiring prior knowledge of adulterant types [38]. This approach is particularly suitable for rapid authenticity identification in complex matrices. In the present study, when the number of PCs was set to seven, the DD-SIMCA model achieved 100% classification accuracy on the test set, indicating that all genuine samples were correctly identified, with no false negatives. Accuracy on the validation set reached 98.2% (Supplementary_Table S3, Figure 3E,F), showing only a slight decrease compared with the test set and indicating that the model generalizes well without evident overfitting.

The discriminant boundary plot further confirmed that all training set samples fell within the model-defined acceptance region, reflecting stable positive-class modeling and strong internal consistency. Only one genuine sample in the test set was misclassified, likely due to minor variations in its spectral features caused by factors such as origin, storage, or processing differences. Nevertheless, this did not affect the overall discriminative performance of the model [39].

Overall, the DD-SIMCA model effectively captures spectral differences between DKM and adulterated samples across key wavebands. It maps high-dimensional spectral data into a low-dimensional feature space via PCA. Even in scenarios with low adulteration ratios or highly deceptive adulterants, the model demonstrates excellent sensitivity and accuracy, highlighting its potential as a robust tool for food authenticity verification and adulteration detection.

### 3.5. Quantitative Analysis Using PLSR

Following the successful identification of DKM adulteration via PCA and machine learning algorithms, a PLSR model was developed to predict adulterant content. To further improve performance, multiple models were constructed, and the effects of different spectral preprocessing methods were systematically evaluated [40]. The optimal preprocessing strategy for each model was primarily determined based on the coefficient of determination (R^2^).

For specific comparisons, the DKM vs. DKML model exhibited optimal performance with SNV preprocessing, achieving an R^2^ of 0.9974. In the DKM vs. corn group, MSC yielded the R^2^ of 0.9992. The DKM vs. bamboo model performed best with the combination of SNV, 1st Der, and SG smoothing, resulting in an R^2^ of 0.9933. For DKM vs. DH, SNV combined with 2nd Der achieved the R^2^ of 0.9687. Finally, in the DKM vs. DL comparison, SNV + 1st Der demonstrated superior performance with an R^2^ of 0.9962.

It should be emphasized that the selection of the optimal preprocessing method was not solely based on R^2^. Model evaluation also considered RMSEC, RMSEP, relative percent deviation (RPD), and mean absolute error (MAE), ensuring that the chosen preprocessing provided high fitting accuracy, minimized prediction errors, and maintained strong generalization. These results highlight that tailoring preprocessing strategies to specific adulterant types significantly enhances the discriminative capability and stability of PLSR models. Overall, SNV and its derivative-based combinations consistently outperformed other preprocessing methods, effectively reducing spectral noise and amplifying differences between genuine and adulterated samples.

**Figure 4:**
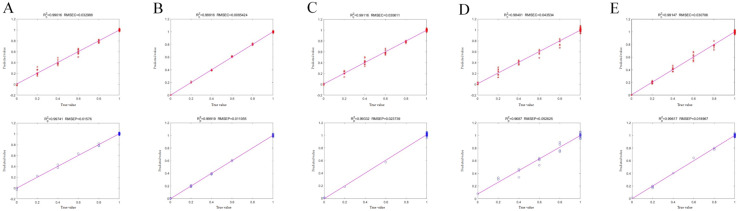
PLSR plots for the optimal models predicting adulterant content in DKM. (**A**) DKM vs. DKML; (**B**) DKM vs. Corn; (**C**) DKM vs. Bamboo; (**D**) DKM vs. DH; (**E**) DKM vs. DL. Data points along the diagonal line indicate agreement between predicted and actual adulteration levels.

The regression plots further illustrate model performance, with data points closely aligned along the diagonal indicate accurate predictions [41] (Figure 4). Specifically, 87.2% of samples exhibited prediction errors ≤0.07%, demonstrating minimal deviation between predicted and actual adulterant concentrations (Table 1). Moreover, the close correspondence between R^2^ and RMSE values in the calibration and prediction sets confirms that the models were not overfit [42].

Collectively, these findings demonstrate that applying targeted preprocessing strategies in PLSR modeling substantially improves quantitative prediction of adulterants in DKM. This approach provides robust support for reliable adulteration detection and accurate concentration analysis.

## 4. Conclusions

In this study, a rapid, non-destructive, and efficient approach for the identification and quantitative analysis of DKM adulteration was established based on NIR spectroscopy combined with chemometric and machine learning methods. PCA and PLS-DA models initially revealed the overall distribution and clustering of samples, while OPLS-DA with orthogonal signal correction improved predictive performance and enhanced discrimination between genuine and adulterated samples.

The DD-SIMCA single-class model demonstrated excellent discriminative performance under the current study conditions. It achieved high-specificity identification of genuine DKM, with accuracies of 100% and 98.2% in the prediction and validation sets, respectively. The model also maintained good sensitivity even at the 20% adulteration ratio. For quantitative analysis, multiple PLSR models targeting different adulterants were constructed. Preprocessing strategies, including SNV, MSC, and derivative transformations, were applied to effectively enhance model fitting and predictive performance.

In conclusion, the integrated NIR–DD-SIMCA–PLSR approach provides a robust and rapid proof-of-concept for the authenticity identification and adulteration quantification of DKM. The non-destructive, high-throughput characteristics of this methodology make it highly promising for its eventual application in the quality control and supervision of DKM products. However, the limited number of genuine batches and laboratory-prepared adulterants may not fully reflect real-world variability, so the high model performance should be interpreted with caution. Future work should focus on expanding sample diversity to include varying origins, processing methods, and commercial contaminants to fully validate its generalizability and transition this powerful method from a preliminary study to broad practical adoption.

## Figures and Tables

**Table 1:** Performance metrics of PLSR models for predicting adulteration content in DKM with different preprocessing methods.

	Methods		Prediction Set				Calibration Set			
		LV ^a^	RMSE	R^2^	MAE	RPD2	RMSE	R^2^	MAE	RPD1
DKM-DKML	1-2+RAW	6.00	0.0316	0.9823	0.0242	7.6363	0.0316	0.9916	0.0224	10.9307
	1-2+1st Der + SG	4.00	0.0262	0.9918	0.0187	11.6183	0.0330	0.9905	0.0240	10.2468
	1-2+1st Der	4.00	0.0259	0.9920	0.0188	11.5929	0.0316	0.9912	0.0231	10.6805
	1-2+2nd Der + SG	2.00	0.0254	0.9932	0.0162	12.1606	0.0332	0.9902	0.0252	10.1186
	1-2+2nd Der	2.00	0.0254	0.9932	0.0162	12.1606	0.0332	0.9902	0.0252	10.1186
	1-2+MSC + 1st Der + SG	2.00	0.0254	0.9932	0.0162	12.1606	0.0332	0.9902	0.0252	10.1186
	1-2+MSC + 1st Der	5.00	0.0181	0.9968	0.0142	17.8033	0.0225	0.9953	0.0179	14.5555
	1-2+MSC + 2nd Der + SG	2.00	0.0190	0.9951	0.0142	15.1223	0.0222	0.9954	0.0175	14.7959
	1-2+MSC + 2nd Der	2.00	0.0152	0.9948	0.0106	14.7168	0.0234	0.9951	0.0184	14.3283
	1-2+MSC	10.00	0.0173	0.9969	0.0133	18.3784	0.0218	0.9957	0.0154	15.2465
	1-2+SNV + 1st Der + SG	5.00	0.0171	0.9972	0.0135	19.8108	0.0245	0.9944	0.0186	13.3339
	1-2+SNV + 1st Der	4.00	0.0287	0.9936	0.0216	12.5349	0.0258	0.9934	0.0185	12.3510
	1-2+SNV + 2nd Der + SG	3.00	0.0168	0.9960	0.0132	17.8962	0.0198	0.9965	0.0157	16.8138
	1-2+SNV + 2nd Der	2.00	0.0122	0.9955	0.0094	16.9292	0.0219	0.9959	0.0172	15.5648
	1-2+SNV	4.00	0.0158	0.9974	0.0119	19.7375	0.0330	0.9902	0.0232	10.0812
DKM--Corn	1-3+RAW	7.00	0.0229	0.9960	0.0194	15.9034	0.0286	0.9918	0.0155	11.0532
	1-3+1st Der + SG	6.00	0.0252	0.9952	0.0175	15.1240	0.0205	0.9958	0.0140	15.3517
	1-3+1st Der	6.00	0.0251	0.9956	0.0180	15.5150	0.0191	0.9962	0.0137	16.1362
	1-3+2nd Der + SG	4.00	0.0243	0.9961	0.0195	16.5934	0.0134	0.9979	0.0108	22.0091
	1-3+2nd Der	3.00	0.0191	0.9976	0.0135	20.9680	0.0169	0.9967	0.0128	17.5060
	1-3+MSC + 1st Der + SG	5.00	0.0130	0.9989	0.0098	30.6277	0.0106	0.9987	0.0080	27.9417
	1-3+MSC + 1st Der	6.00	0.0225	0.9968	0.0186	18.9177	0.0156	0.9971	0.0116	18.5735
	1-3+MSC + 2nd Der + SG	4.00	0.0251	0.9956	0.0212	15.1725	0.0144	0.9975	0.0110	19.9671
	1-3+MSC + 2nd Der	11.00	0.1121	0.6089	0.0919	1.6416	0.0399	0.9883	0.0323	9.2358
	1-3+MSC	12.00	0.0111	0.9992	0.0088	35.1258	0.0085	0.9992	0.0069	34.4660
	1-3+SNV + 1st Der + SG	7.00	0.0235	0.9965	0.0183	17.1097	0.0164	0.9968	0.0120	17.6387
	1-3+SNV + 1st Der	6.00	0.0233	0.9965	0.0175	19.2068	0.0169	0.9966	0.0126	17.1283
	1-3+SNV + 2nd Der + SG	3.00	0.0575	0.9707	0.0423	6.1333	0.0312	0.9910	0.0242	10.5476
	1-3+SNV + 2nd Der	3.00	0.0778	0.9489	0.0523	5.1688	0.0224	0.9953	0.0182	14.5359
	1-3+SNV	10.00	0.0155	0.9984	0.0121	26.0146	0.0149	0.9975	0.0106	19.8187
DKM-Bamboo	1-4+RAW	3.00	0.0333	0.9739	0.0255	6.9138	0.0563	0.9729	0.0432	6.0769
	1-4+1st Der + SG	5.00	0.0376	0.9837	0.0306	7.8277	0.0403	0.9849	0.0318	8.1389
	1-4+1st Der	5.00	0.0365	0.9846	0.0291	8.0673	0.0369	0.9873	0.0287	8.8858
	1-4+2nd Der + SG	3.00	0.0349	0.9856	0.0284	9.2994	0.0426	0.9832	0.0326	7.7030
	1-4+2nd Der	3.00	0.0350	0.9876	0.0275	10.5942	0.0373	0.9868	0.0287	8.7168
	1-4+MSC + 1st Der + SG	6.00	0.0241	0.9931	0.0192	12.2418	0.0300	0.9915	0.0222	10.8633
	1-4+MSC + 1st Der	6.00	0.0246	0.9928	0.0203	12.3013	0.0274	0.9929	0.0203	11.8895
	1-4+MSC + 2nd Der + SG	4.00	0.0345	0.9880	0.0282	9.2221	0.0259	0.9937	0.0195	12.5491
	1-4+MSC + 2nd Der	3.00	0.0382	0.9742	0.0331	7.0985	0.0325	0.9906	0.0254	10.3276
	1-4+MSC	3.00	0.0233	0.9917	0.0184	11.2458	0.0460	0.9811	0.0344	7.2671
	1-4+SNV + 1st Der + SG	6.00	0.0237	0.9933	0.0187	13.2774	0.0306	0.9912	0.0225	10.6384
	1-4+SNV + 1st Der	3.00	0.0242	0.9432	0.0191	4.3693	0.0388	0.9873	0.0293	8.8738
	1-4+SNV + 2nd Der + SG	3.00	0.0354	0.9873	0.0282	9.2963	0.0339	0.9891	0.0269	9.5705
	1-4+SNV + 2nd Der	6.00	0.0455	0.9803	0.0324	7.1181	0.0061	0.9997	0.0050	54.8534
	1-4+SNV	4.00	0.0248	0.9906	0.0199	10.7403	0.0455	0.9815	0.0337	7.3464
DKM-DH	1-5+RAW	5.00	0.0867	0.9337	0.0689	3.9678	0.0862	0.9309	0.0561	3.8049
	1-5+1st Der + SG	5.00	0.0770	0.9504	0.0559	4.7411	0.0677	0.9563	0.0522	4.7833
	1-5+1st Der	4.00	0.0670	0.9625	0.0539	5.4756	0.0710	0.9521	0.0540	4.5671
	1-5+2nd Der + SG	3.00	0.0602	0.9680	0.0456	5.6123	0.0662	0.9593	0.0485	4.9574
	1-5+2nd Der	4.00	0.0579	0.9675	0.0428	5.7525	0.0392	0.9861	0.0302	8.4889
	1-5+MSC + 1st Der + SG	5.00	0.0811	0.9404	0.0678	4.1180	0.0649	0.9602	0.0514	5.0111
	1-5+MSC + 1st Der	5.00	0.0777	0.9450	0.0621	4.2652	0.0595	0.9661	0.0486	5.4340
	1-5+MSC + 2nd Der + SG	2.00	0.0426	0.9647	0.0320	5.6658	0.0738	0.9544	0.0525	4.6806
	1-5+MSC + 2nd Der	2.00	0.0467	0.9575	0.0356	5.0981	0.0707	0.9581	0.0495	4.8846
	1-5+MSC	8.00	0.0566	0.9649	0.0424	5.5307	0.0694	0.9569	0.0514	4.8162
	1-5+SNV + 1st Der + SG	5.00	0.0729	0.9451	0.0603	4.3693	0.0630	0.9637	0.0495	5.2470
	1-5+SNV + 1st Der	5.00	0.0784	0.9435	0.0621	4.4402	0.0553	0.9714	0.0433	5.9144
	1-5+SNV + 2nd Der + SG	3.00	0.0760	0.9491	0.0608	4.6551	0.0457	0.9807	0.0354	7.1903
	1-5+SNV + 2nd Der	3.00	0.0526	0.9687	0.0412	5.9448	0.0435	0.9840	0.0331	7.9074
	1-5+SNV	8.00	0.0631	0.9563	0.0455	4.8122	0.0727	0.9527	0.0519	4.5992
DKM-DL	1-6+RAW	6.00	0.0399	0.9883	0.0315	9.5610	0.0439	0.9803	0.0350	7.1253
	1-6+1st Der + SG	5.00	0.0413	0.9882	0.0312	9.3527	0.0317	0.9890	0.0260	9.5512
	1-6+1st Der	4.00	0.0438	0.9867	0.0326	8.8247	0.0362	0.9857	0.0280	8.3595
	1-6+2nd Der + SG	4.00	0.0512	0.9822	0.0425	8.0835	0.0205	0.9954	0.0160	14.7521
	1-6+2nd Der	3.00	0.0443	0.9857	0.0333	8.8207	0.0275	0.9922	0.0224	11.3274
	1-6+MSC + 1st Der + SG	4.00	0.0242	0.9940	0.0200	13.0082	0.0280	0.9926	0.0213	11.6028
	1-6+MSC + 1st Der	5.00	0.0237	0.9946	0.0190	13.7550	0.0226	0.9952	0.0172	14.3819
	1-6+MSC + 2nd Der + SG	3.00	0.0255	0.9900	0.0197	11.1332	0.0199	0.9965	0.0157	16.9278
	1-6+MSC + 2nd Der	2.00	0.0179	0.9573	0.0143	4.8529	0.0335	0.9906	0.0261	10.3108
	1-6+MSC	6.00	0.0283	0.9923	0.0187	11.4111	0.0326	0.9901	0.0244	10.0460
	1-6+SNV + 1st Der + SG	5.00	0.0216	0.9955	0.0171	15.0779	0.0229	0.9950	0.0174	14.1917
	1-6+SNV + 1st Der	3.00	0.0190	0.9962	0.0156	16.1756	0.0307	0.9915	0.0231	10.8291
	1-6+SNV + 2nd Der + SG	3.00	0.0220	0.9940	0.0170	14.1099	0.0190	0.9968	0.0147	17.5750
	1-6+SNV + 2nd Der	3.00	0.0176	0.9520	0.0130	4.6557	0.0173	0.9975	0.0144	19.8540
	1-6+SNV	5.00	0.0293	0.9931	0.0230	12.0693	0.0385	0.9853	0.0321	8.2344

Notes: LV ^a^: Latent Variable.

## Data Availability

The original contributions presented in the study are included in the article; further inquiries can be directed to the corresponding author.

## Supplementary Materials

The following supporting information can be downloaded at https://www.mdpi.com/article/10.3390/foods15010121/s1, Supplementary_Material S1–S3 and Supplementary Figure S1.

## Abbreviations

The following abbreviations are used in this manuscript:

NIR	Near-Infrared
LIBS	Laser-Induced Breakdown Spectroscopy
DD-SIMCA	Data-Driven Soft Independent Modeling of Class Analogy
PLSR	Partial Least Squares Regression
MSC	Multiplicative Scatter Correction
SG	Savitzky–Golay
SNV	Standard Normal Variate Transformation
DKM	Dendrobium officinale Kimura et Migo
DL	Dendrobium nobile Lindl
DH	Dendrobium fimbriatum Hook
DKML	DKM leaves
PCA-X	Principal Component Analysis
PLS-DA	Partial Least Squares Discriminant Analysis
OPLS-DA	Orthogonal Partial Least Squares Discriminant Analysis
DT	Decision Tree
SVM	Support Vector Machine
ANN	Artificial Neural Network
NB	Naive Bayes
LVs	Latent Variables
RMSECV	Root Mean Square Error of Cross Validation
RMSEC	Root Mean Square Error of Calibration
RMSEP	Root Mean Square Error of Prediction
MAE	Mean Absolute Error
RPD	Relative Percent Deviation
1st Der	First Derivative
PCs	Principal Components

## References

[1] Wang M., Shao G., Song M., Ye Y., Zhu J., Yang X., Song X. (2025). Dynamic Changes in Functional Components of *Dendrobium officinale* and Their Applications in Food Science: A Review. Plant Foods Hum. Nutr..

[2] Li P.Y., Li L., Wang Y.Z. (2023). Traditional uses, chemical compositions and pharmacological activities of Dendrobium: A review. J. Ethnopharmacol..

[3] Qu J., Tan S., Xie X., Wu W., Zhu H., Li H., Liao X., Wang J., Zhou Z.-A., Huang S. (2021). *Dendrobium officinale* Polysaccharide Attenuates Insulin Resistance and Abnormal Lipid Metabolism in Obese Mice. Front. Pharmacol..

[4] Duan H., Yu Q., Ni Y., Li J., Yu L., Yan X., Fan L. (2024). Synergistic anti-aging effect of *Dendrobium officinale* polysaccharide and spermidine: A metabolomics analysis focusing on the regulation of lipid, nucleotide and energy metabolism. Int. J. Biol. Macromol..

[5] Li K., Guo Y., Zhong H., Jin Y., Li B., Fang H., Yao L., Zhao C. (2025). Rapid identification of dendrobium species using near-infrared hyperspectral imaging technology. Sensors.

[6] Zhang T., Liu Z., Ma Q., Hu D., Dai Y., Zhang X., Zhou Z. (2024). Identification of dendrobium using laser-induced breakdown spectroscopy in combination with a multivariate algorithm model. Foods.

[7] Lin T., Ye Y., Zhang J., Wang J., Hu Z., Linn K.Z., Chen X., Liu H., Liu Z., Yao Q. (2025). Machine learning and uhplc-ms/ms-based discrimination of the geographical origin of *Dendrobium officinale* from Yunnan, China. Foods.

[8] Zhang X., Zhang S., Gao B., Qian Z., Liu J., Wu S., Si J. (2019). Identification and quantitative analysis of phenolic glycosides with antioxidant activity in methanolic extract of *Dendrobium catenatum* flowers and selection of quality control herb-markers. Food Res. Int..

[9] Deng Y., Chen L.-X., Han B.-X., Wu D.-T., Cheong K.-L., Chen N.-F., Zhao J., Li S.-P. (2016). Qualitative and quantitative analysis of specific polysaccharides in *Dendrobium huoshanense* by using saccharide mapping and chromatographic methods. J. Pharm. Biomed. Anal..

[10] Qin H.L., Zhang J.X., Wang Z.T., Yang X.S., Xu L.S., Hao X.J. (2002). Analysis of 1H-NMR fingerprint in stem of *Dendrobium loddigesii*. Zhongguo Zhong Yao Za Zhi.

[11] Hao L., Shi X., Qin S., Dong J., Shi H., Wang Y., Zhang Y. (2023). Genome-wide identification, characterization and transcriptional profile of the SWEET gene family in *Dendrobium officinale*. BMC Genom..

[12] Meng Y., Wang Y., Zhang L., Li J., Hu L., Wu Z., Yang L., Wei G. (2023). Identification of bibenzyls and evaluation of imitative wild planting techniques in *Dendrobium officinale* by HPLC-ESI-MS(n). J. Mass Spectrom. JMS.

[13] Bec K.B., Grabska J., Huck C.W. (2020). Near-Infrared Spectroscopy in Bio-Applications. Molecules.

[14] Yu Y., Chai Y., Yan Y., Li Z., Huang Y., Chen L., Dong H. (2025). Near-infrared spectroscopy combined with support vector machine for the identification of Tartary buckwheat (*Fagopyrum tataricum* (L.) Gaertn) adulteration using wavelength selection algorithms. Food Chem..

[15] Guan H., Zhang Z.T., Bai L., Chen L., Yuan D., Liu W., Chen P., Shi Z., Hu C., Xue M. (2024). Multi-spectra combined with Bayesian optimized machine learning algorithms for rapid and non-destructive detection of adulterated functional food *Panax notoginseng* powder. J. Food Compos. Anal..

[16] Li X., Zhong Y., Li J., Lin Z., Pei Y., Dai S., Sun F. (2024). Rapid identification and determination of adulteration in medicinal Arnebiae Radix by combining near infrared spectroscopy with chemometrics. Spectrochim. Acta Part A Mol. Biomol. Spectrosc..

[17] Ben Salem K., Ben Abdelaziz A. (2021). Principal Component Analysis (PCA). Tunis. Med..

[18] Baddini A.L.Q., Santos J., Tavares R.R., Paula L.S., Filho H., Freitas R.P. (2022). PLS-DA and data fusion of visible Reflectance, XRF and FTIR spectroscopy in the classification of mixed historical pigments. Spectrochim. Acta Part A Mol. Biomol. Spectrosc..

[19] Becker T., Rousseau A.J., Geubbelmans M., Burzykowski T., Valkenborg D. (2023). Decision trees and random forests. Am. J. Orthod. Dentofac. Orthop..

[20] Rodriguez-Perez R., Bajorath J. (2022). Evolution of Support Vector Machine and Regression Modeling in Chemoinformatics and Drug Discovery. J. Comput.-Aided Mol. Des..

[21] Jirik M., Moulisova V., Hlavac M., Zelezny M., Liska V. (2022). Artificial neural networks and computer vision in medicine and surgery. Rozhl. Chir. Mesic. Ceskoslovenske Chir. Spol..

[22] Fautt C., Couradeau E., Hockett K.L. (2024). Naive Bayes Classifiers and accompanying dataset for *Pseudomonas syringae* isolate characterization. Sci. Data.

[23] Pagani A.P., Camargo G., Ibanez G.A., Olivieri A.C., Pomerantsev A.L., Rodionova O.Y. (2024). Data-Driven Version of Multiway Soft Independent Modeling of Class Analogy (N-Way DD-SIMCA): Theory and Application. Anal. Chem..

[24] Jin C., Zhou X., He M., Li C., Cai Z., Zhou L., Qi H., Zhang C. (2024). A novel method combining deep learning with the Kennard-Stone algorithm for training dataset selection for image-based rice seed variety identification. J. Sci. Food Agric..

[25] Walston S.L., Seki H., Takita H., Mitsuyama Y., Sato S., Hagiwara A., Ito R., Hanaoka S., Miki Y., Ueda D. (2024). Data set terminology of deep learning in medicine: A historical review and recommendation. Jpn. J. Radiol..

[26] Sun W., Liu S., Zhang X., Zhu H. (2022). Performance of hyperspectral data in predicting and mapping zinc concentration in soil. Sci. Total Environ..

[27] Liu Z., Zhang R., Yang C., Hu B., Luo X., Li Y., Dong C. (2022). Research on moisture content detection method during green tea processing based on machine vision and near-infrared spectroscopy technology. Spectrochim. Acta Part A Mol. Biomol. Spectrosc..

[28] Wu L., Su Y., Yu H., Qian X., Zhang X., Wang Q., Kuang H., Cheng G. (2018). Rapid Determination of Saponins in the Honey-Fried Processing of Rhizoma Cimicifugae by Near Infrared Diffuse Reflectance Spectroscopy. Molecules.

[29] Kang Y., Long T., Qiao Y., Yi H., Wang F., Chen C. (2025). Rapid quality evaluation of fried Radix Paeoniae Alba (*Paeonia lactiflora* Pall.) using electronic eye and near-infrared spectroscopy combined with chemometric methods. J. Food Compos. Anal..

[30] Fang H., Wang Y., Deng J., Zhang H., Wu Q., He L., Xu J., Shao X., Ouyang X., He Z. (2022). Sepsis-Induced Gut Dysbiosis Mediates the Susceptibility to Sepsis-Associated Encephalopathy in Mice. mSystems.

[31] Fu S., Liu F., Zhi X., Wang Y., Liu Y., Chen H., Wang Y., Luo M. (2023). Applications of functional near-infrared spectroscopy in non-drug therapy of traditional Chinese medicine: A review. Front. Neurosci..

[32] Yan C. (2025). A review on spectral data preprocessing techniques for machine learning and quantitative analysis. iScience.

[33] Zhang B., Chen X., He C., Su T., Cao K., Li X., Duan J., Chen M., Zhu Z., Yu W. (2023). Acute gastrointestinal injury and altered gut microbiota are related to sepsis-induced cholestasis in patients with intra-abdominal infection: A retrospective and prospective observational study. Front. Med..

[34] Gao Z., Zhang M., Liu N., Liang W., Sun T. (2025). Distinguishing Low-Grade Chondrosarcoma and Osteochondroma Using Visible-Near Infrared Hyperspectral Spectral Characteristics. J. Biophotonics.

[35] Alsariera Y.A., Baashar Y., Alkawsi G., Mustafa A., Alkahtani A.A., Ali N. (2022). Assessment and Evaluation of Different Machine Learning Algorithms for Predicting Student Performance. Comput. Intell. Neurosci..

[36] Bao H., Bao H., Wang Y., Wang F., Jiang Q., He X., Li H., Ding Y., Zhu C. (2024). Challenges and Strategies in the Industrial Application of *Dendrobium officinale*. Plants.

[37] Rodionova O., Pomerantsev A. (2023). Multi-block DD-SIMCA as a high-level data fusion tool. Anal. Chim. Acta.

[38] de Sousa J.F., Batista Braga J.W., Dias A.C.B. (2025). Authenticity assessment of commercial natural sweeteners using near- and mid-infrared spectroscopy with DD-SIMCA modeling. Food Chem..

[39] Candeias D.N.C., Silva K.M., Pereira H.S., Bezerra L.P., da Silva J.D.S., Fernandes D.D.S., Diniz P.H.G.D. (2025). Geographical origin authentication of instant coffee from southern Bahia using MIR and NIR spectroscopy coupled with DD-SIMCA. Food Chem..

[40] Lan Z., Zhang Y., Sun Y., Ji D., Wang S., Lu T., Cao H., Meng J. (2021). Rapid quantitative detection of the discrepant compounds in differently processed Curcumae Rhizoma products by FT-NIR combined with VCPA-GA technology. J. Pharm. Biomed. Anal..

[41] Bai L., Zhang Z.-T., Guan H., Liu W., Chen L., Yuan D., Chen P., Xue M., Yan G. (2024). Rapid and accurate quality evaluation of Angelicae Sinensis Radix based on near-infrared spectroscopy and Bayesian optimized LSTM network. Talanta.

[42] Daba S.D., Honigs D., McGee R.J., Kiszonas A.M. (2022). Prediction of Protein Concentration in Pea (*Pisum sativum* L.) Using Near-Infrared Spectroscopy (NIRS) Systems. Foods.

